# Modification of the TRP Channel TRPA1 as a Relevant Factor in Migraine-Related Intracranial Hypersensitivity

**DOI:** 10.3390/ijms24065375

**Published:** 2023-03-11

**Authors:** Thannoon Masood, Szandra Lakatos, Judit Rosta

**Affiliations:** 1 Department of Neurosurgery, Albert Szent-Györgyi Medical School, University of Szeged, 6725 Szeged, Hungary; 2 Department of Physiology, Albert Szent-Györgyi Medical School, University of Szeged, 6720 Szeged, Hungary

**Keywords:** TRPA1, migraine, hypersensitivity

## Abstract

Recently, the transient receptor potential ankyrin 1 (TRPA1) has gained more attention in migraine-related research. The involvement of the TRPA1 receptor in migraine headaches is proposed by the fact that TRPA1 may be a target of some migraine-triggering factors. Although it is doubtful that activation of TRPA1 alone is sufficient to induce pain, behavioral studies have demonstrated that TRPA1 is involved in injury- and inflammation-induced hypersensitivity. Here, we review the functional relevance of TRPA1 in headaches and its therapeutic potential, mainly focusing on its role in the development of hypersensitivity, referring to its altered expression in pathological conditions, and its functional interaction with other TRP channels.

## 1. Introduction

Migraine is the most common type of primary headache and is characterized by regularly recurring headache attacks, often associated with other neurological symptoms and increased sensitivity, such as mechanical allodynia. Many environmental irritants have been identified as headache-provoking factors.

The involvement of transient receptor potential ankyrin 1 (TRPA1) in the sensation of pain induced by irritants has been widely confirmed by functional and behavioral studies. Several chemical irritants and pain-inducing factors of different natures can induce TRPA1 activation, suggesting that TRPA1 is a common target of variously initiated pain pathways. In the last decade, the TRPA1 receptor has become a putative target as a headache-provoking factor. The results of some currently ongoing clinical trials have not been reported, so the analgesic potency of TRPA1 inhibition is not yet fully elucidated.

Several studies have already pointed out the relevance of TRPA1 in the pathomechanism of pain sensation and migraine. In this review, we focus on the importance of a possible functional modification of TRPA1 in the pathomechanism of migraine and associated hypersensitivity.

### 1.1. Migraine

Head pain and migraine-associated research is a marginal but notable field of pain investigation. As the most common chronic headache disorder, migraine affects more than 10% of the population [1]. Despite intensive migraine research, the pathomechanisms of migraine and other primary headaches are still unclear.

Migraine is a complex neurological disorder characterized by throbbing head pain, typically localized to one side of the head [2]. Migraine is often accompanied by diverse vegetative and sensory symptoms such as nausea, vomiting, and increased sensitivity to light and sound [3,4]. The recurring headache attacks, with intense pain sensations localized to the head and neck region, can last from hours to days. Chronic migraine is frequently associated with other neurological symptoms, e.g., visual disturbances such as scotoma, blurred vision, and disturbed contours of objects—taken together called a ‘visual aura’ [5]. The phenomenon of aura is also applied to other sensory disturbances, such as increased peripheral pain sensation, paresthesia, dysphasia, or olfactory hallucinations [6]. According to different studies, 10–12% of the population is affected, and two-thirds of migraine patients are women [7,8]. Due to migraine’s high prevalence and financial burden, pain research studies have made a huge effort for decades to reveal the underlying mechanisms. In spite of intensive research and a large number of clinical studies, the pathomechanism of migraine, however, has not yet been clearly revealed.

Experimental migraine studies mainly focus on developing intracranial pain as the most readily detectable symptom of migraine. Headaches categorized as primary, such as migraine, cluster- or tension-type headaches, are classified based on the absence of any specific underlying reason [9,10]. Specifically, pain sensitivity is restricted primarily to the blood vessels of the meninges, such as the dura mater encephali, pia mater, and the major cerebral vessels. The sensory innervation of these structures is raised from the trigeminal nerve. It is well-established that the activation of trigeminal nerves is closely related to head pain [11]. The trigeminovascular system involving primary sensory neurons in the trigeminal ganglion (TG), the trigeminal afferents, and their target structures such as meningeal and cerebral blood vessels [12], as well as the spinal trigeminal nuclei, remains a major focus of current concepts regarding the origins of head pain.

#### Migraine-Associated Hypersensitivity

A typical migraine-related symptom is that, initially, non-painful stimuli, e.g., small head movements, become painful during headache attacks. This phenomenon is called intracranial hypersensitivity, which commonly accompanies headache periods and is a characteristic symptom in primary headache disorders.

Trigeminal activation results in neurogenic inflammation, a well-known component of the pathogenesis of migraine [13]. A neurogenic response is characterized by mast cell activation, vasodilation, macrophage activation, and plasma extravasation. Using imaging techniques, increased vascular permeability and inflammatory activity have been detected during a migraine attack [14,15]. Migraine-associated hypersensitivity is known to be a consequence of the release of inflammatory mediators, including prostaglandins and prostacyclin.

This heightened sensitivity manifests in migraine-associated mechanical allodynia, suggesting that the pain processing mechanisms may have been altered. The accompanying reduced pain threshold raises the question of how the function of intracranial nociceptors changes during migraine attacks. The concept of ‘silent nociceptors’ refers to the existence of high-threshold afferents that do not respond to moderate mechanical stimuli and become mechanosensitive only when exposed to prolonged noxious stimuli. According to the concept, the silent nociceptors may become responsive, e.g., during inflammation, due to the presence of inflammatory mediators [16]. Presumably, these silent afferents also exist in the tissues of the meninges. Burstein et al. found a subpopulation of meningeal sensory fibers that initially showed little or no response to mechanical stimuli; however, they became mechanically sensitive after inflammatory agents were applied to the surface of the dura [17]. That result proved that long-term chemical stimulation results in sensitization, meaning a reduced threshold of intracranial afferents to mechanical stimuli.

Similarly to inflammatory mediators, inhalation of the TRPA1 channel agonist acrolein was found to cause such sensitization of meningeal afferents by stimulating nasal axon collaterals, thereby increasing the risk of migraine attacks in susceptible individuals [18].

### 1.2. Transient Receptor Potential Ankyrin 1 (TRPA1)

In the last two decades, members of the TRP (transient receptor potential) superfamily have been identified to play a pivotal role in peripheral nociception and the sensation of pain [19,20,21,22]. TRPA1—formerly known as ankyrin-like with transmembrane domains protein 1 [ANKTM1], originally described by Story et al. [23]—is expressed by a small subpopulation of dorsal root ganglion neurons, as well as trigeminal neurons [24,25]. TRPA1 is activated by various noxious compounds such as allyl isothiocyanate (AITC), the pungent agent of mustard oil (MO), horseradish, and cinnamaldehyde [26,27]. Acrolein, an irritant compound found in tear gas and cigarette smoke, was the first substance to be identified as a ‘specific TRPA1 activator’, and, furthermore, electrophilic substances such as Δ9-tetrahydrocannabinol, icilin, and 2-aminoethoxydiphenyl borate (2APB) were also cited as TRPA1 activators [26,28].

The functional importance of the TRPA1 channel in peripheral pain sensation is suggested by the fact that TRPA1 expression is typically attributed to the small-sized neurons, commonly referred to as nociceptors. TRPA1-mediated pain sensation was reported in subjects following the administration of a synthetic TRPA1 agonist, suggesting that TRPA1 activation alone is sufficient to evoke pain in patients [29]. In addition, the importance of TRPA1 in human pain sensation is indicated by a genetic disorder in which TRPA1 is affected. Familial episodic pain syndrome, a genetic disorder accompanied by episodic body pain, has been shown to be associated with a TRPA1 deficiency [30].

#### Emphasized Role of TRPA1 in Hypersensitivity Reactions

Originally, TRPA1 transcripts were found in thermo- and mechanosensory neurons; therefore, TRPA1 was cited as the missing link in cold-induced (<17 °C) pain sensation [23]. However, an increasing body of evidence suggests that TRPA1 is not necessary for cold-induced pain and that cold-associated nociception is not mediated by TRPA1 but requires other TRP channels [31]. Although the pain-inducing effect of TRPA1 activation has not yet been satisfactorily proven, the relevance of TRPA1 in inflammation and irritant-induced hypersensitivity is strongly confirmed.

Nearly two decades ago, TRPA1 was reported to be involved in inflammation-induced hyperalgesia [32]. This study also showed that TRPA1 expression is increased in sensory neurons following both inflammation and nerve injury. The functional relevance of the TRPA1 channel in hypersensitivity is supported by the fact that the upregulation of TRPA1 is also associated with experimental models of neuropathic and inflammation-induced pain [29,33,34].

Most of the above-mentioned chemical irritants can activate TRPA1 receptors by covalent modification of certain cysteine residues of the protein [25]. In addition, agents that stimulate phospholipase C appear to indirectly activate TRPA1. In this way, TRPA1 receptors are also involved in neuronal responses triggered by the inflammatory mediators prostaglandin E2, bradykinin, or acetaldehyde [35,36]. It has been demonstrated that since cold-induced pain was preserved, bradykinin-evoked thermal hyperalgesia was diminished in TRPA1 knockout mice [37]. TRPA1 is also involved in the transducing mechanisms of the cough-sensitizing actions of bradykinin. Bradykinin has been shown to induce the sensitization of second-order neurons via the release of cyclooxygenase and 12-lipoxygenase metabolites, which can activate TRPA1 [38]. Also, spinal nerve ligation-induced cold hyperalgesia is decreased by TRPA1 knock down [39]. Recently, hyperalgesia and allodynia induced by certain itch mediators have also been shown to be mediated by TRPA1 [40].

Chemotherapy-induced peripheral neuropathy is characterized by allodynia and hyperalgesia. Anticancer drugs such as carboplatin or oxaliplatin have been shown to evoke mechanical allodynia and cold hyperalgesia through TRPA1 activation [41,42]. In neuropathic pain models, the modification of the TRPA1 channel seems to be involved in the development of hypersensitivity. The sensitization of the TRPA1 channel via the cyclic adenosine monophosphate–protein kinase A pathway has been demonstrated [41]. The ubiquitination of the TRPA1 channel by the neural precursor cell-expressed developmentally downregulated protein 4-2 (Nedd4-2), which is synthesized by sensory neurons, has been described. Wang et al. reported a disturbed expression of Nedd4-2 in a diabetic mouse model and hypothesized that modification of TRPA1 contributes to diabetic neuropathic pain [43].

Interganglionic transmission, which emphasizes the importance of communication between neurons and non-neuronal glial cells, provides another aspect of peripheral hypersensitivity. A recent study showed that non-neuronal satellite glial cells in dorsal root ganglia also express TRPA1 channels, which become more sensitive following inflammation [44]. Neuroinflammation and the related activation of glial cells are involved in the pathophysiology of migraine. The knockout of TRPA1 results in a change in the glial phenotype, suggesting that TRPA1 plays a relevant role in neuroinflammation [45]. A recent study showed that TRPA1 contributes to glial activation in a chronic migraine model [46].

### 1.3. TRPA1 in Migraine-Related Research

#### 1.3.1. The Presence of TRPA1 in the Trigeminal System

The migraine-inducing effect of environmental irritants has been associated with the activity of certain pain-related receptors over time. In recent decades, the classical TRP channel TRPV1, the mechanosensor Piezo channels, or the purinergic receptor PY have been identified as potential targets of certain migraine-triggering substances. In 2011, when Kunkler et al. reported TRPA1-mediated vasodilation in meningeal vessels, TRPA1 came into the focus of migraine-related research [47]. As TRPA1 has gained more attention in relation to head pain, the ratio of the TRPA1-expressing neurons in the intracranial sensory system involving the TG has become the subject of investigation. By using an in situ hybridization technique, it was found that TRPA1 mRNA is expressed in 36.5% of all trigeminal primary sensory neurons [48]. Similar results are obtained by functional studies, as the application of the TRPA1 agonist AITC stimulates nearly 35% of rat trigeminal neurons [26]. TG is responsible for the sensory innervation of the entire head region, including the facial skin, nasal mucosa, intracranial structures, meningeal vasculature, etc. Focusing on head pain, the neurons responsible for innervating the pain-sensitive structures within the skull must be distinguished from the overall population. Applying whole-cell patch-clamp recording, approximately 40% of the dural projection neurons responded to the application of the TRPA1 agonist MO, representing a slightly higher proportion than the total trigeminal neuronal population [49]. This result supports the substantial role of TRPA1 in intracranial sensory processes, as more than one-third of primary sensory neurons—considered potential pain-sensitive neurons—are involved in TRPA1-mediated reactions. Interestingly, only a small percentage of trigeminal neurons projecting to the dura show TRPA1-immunoreactivity [50]. However, the TRPA1-immunoreactive neurons cluster around most of the dural projection neurons, suggesting potential cross-excitation between the neighboring neurons.

The release of CGRP and the resulting vasodilation are highly associated with chronic headache [51,52,53]. CGRP-release from dural afferents is a commonly used model for head pain, supporting the relevance of a substance in trigeminovascular responses associated with headaches. The release of axonal CGRP in a TRPA1-dependent manner has already been demonstrated in a tracheal preparation and the skin [54,55]. In TG-derived neuronal cell cultures, the TRPA1 agonist acrolein has been found to induce the release of CGRP [47]. A direct role of TRPA1 in vascular reactions mediated by trigeminal afferents was recently confirmed by an in vivo study, which demonstrated CGRP release and meningeal vasodilation after TRPA1 activation [56].

An interesting recent study suggests a distinct connection between the activation of TRPA1 and the expression of CGRP. The activation of TRPA1 modulated the CGRP expression and led to an increase in the number of CGRP-immunopositive neurons in a DRG cell culture [57]. The significance of this new result is not yet clear; however, an altered expression of CGRP may be particularly meaningful with regard to headaches. A recent study demonstrated that an increased expression of CGRP in the trigeminal ganglion is mediated by TRPA1 in both acute and chronic migraine models [46].

#### 1.3.2. Relevance of TRPA1 in Migraine-Like Pain

NO was the first experimentally confirmed migraine-provoking factor, and its experimental administration has since been used as a widely accepted migraine model [58]. Tear gas was also considered a headache trigger, as in 29% of seizure events, patients complained about post-seizure headaches [59]. In recent decades, several natural compounds have been identified as headache-provoking factors: camphor, tear gas, formalin, cigarette smoke, or alcoholic beverages—all these agents are well-known headache triggers [60]. TRPA1 was originally identified as a ‘nociceptive’ receptor activated by thermal and mechanical stimuli; however, more and more headache-inducing factors have also been revealed to be TRPA1 activators. Several migraine triggers, such as formalin, cigarette smoke, and even tear gas, have been shown to act through the TRPA1 channel [47,61,62,63]. Moreover, the classical trigger, nitric oxide (NO), has also been found to induce pain sensations through the activation of TRPA1 [64].

In addition, an endogenous product, 4-hydroxynonenal, which is associated with several chronic diseases, has been shown to act as a potential activator of TRPA1, supporting the relevance of TRPA1-activation in chronic pain disorders such as migraine [65].

All of the above studies confirmed that certain migraine-inducing agents are potential activators of TRPA1; however, they did not unambiguously establish a direct link between the headache and TRPA1 receptor activation. It has recently been shown that TRPA1 contributes to migraine-associated hypersensitivity, since the TRPA1 antagonist, ADM_12, prevented the nitroglycerine-induced hyperalgesia in both the acute and chronic migraine models [46].

It was recognized early that headache attacks are accompanied by intracranial vascular reactions [66,67]. Vascular events were originally thought to cause the sensation of pain during headache attacks. However, the direct relationship between head pain and vascular reactions was disproved by modern imaging studies two decades ago [68]. At the same time, meningeal vasodilation is a clear consequence of the activation of chemosensitive trigeminal afferents, so the monitoring of intracranial vascular reactions following trigeminal nerve stimulation serves as an approved model for migraine.

The phenomenon that inhalation of environmental irritants frequently triggers a migraine attack has led researchers to investigate the functional connection between nasal stimulation and headache-related meningeal events. The intranasal administration of chemical compounds and recording the evoked meningeal reactions provide a specific aspect of headache research. The putative role of TRPA1 in intracranial sensitization is supported by Kunkler et al. [47]. A nasal application of the TRPA1 activators MO and acrolein has been shown to increase the meningeal blood flow in a CGRP-dependent way. Subsequently, this group also demonstrated the sensitizing effect of TRPA1 activation on trigeminovascular responses, as chronic exposure to acrolein enhances TRPA1-mediated meningeal vasodilation and leads to facial allodynia [18]. In 2012, an experimental study showed that activation of TRPA1 via an injected dural cannula increased the pain sensation in the facial region [49]. Another behavioral study proved that umbellulone (UMB), the active compound of Umbellularia californica (‘headache tree’), causes trigeminal pain via TRPA1 [69]. It found that in 40% of rat trigeminal ganglion neurons, the administration of UMB could stimulate TRPA1, and intranasal and intravenous administration induced nociceptive behavior in a TRPA1-dependent manner

Cortical spreading depression (CSD) is an electrical disturbance of cortical neurons that spreads along the cortical surface and is accompanied by variable vascular events. CSD is thought to be related to the aura phenomenon of migraine, and, therefore, experimentally triggered CSD events also serve as an accepted migraine model. It was shown that the commonly used TRPA1 agonist AITC and umbellulone also promoted the spread of CSD, indicating not only the involvement of TRPA1 in migraine with aura but also the sensitizing act of TRPA1 activation in the pathomechanism of the aura phenomenon [70].

A recent study reported an altered synthesis of trigeminal TRPA1 triggered by chronic exposure to acrolein and suggests a key role of this process in the chronification of migraine [71]. The special role of TRPA1 in the sensory functions of the meninges is emphasized by the fact that increased TRPA1 mRNA levels were found in the trigeminal neurons projecting to the meninges, but not in nasal trigeminal or dorsal root ganglion neurons.

### 1.4. Possible Cooperation between TRPA1 and TRPV1

It is well-documented that other classical TRP channels, e.g., the transient receptor potential vanilloid 1 (TRPV1), have a pivotal role in inflammation-related hypersensitivity such as thermal hyperalgesia and allodynia [72,73,74]. Hypersensitivity can develop as a result of a functional modification of the TRPV1 by phosphorylation-induced sensitization [75,76,77]. Considering the importance of intracranial sensitization in the pathomechanism of migraine, the functional sensitization of TRPA1, TRPV1, and other TRP receptors involved in intracranial pain sensation at the receptor level should be discussed as a provocative factor in migraine attacks.

Based on its structure, TRPA1 belongs to the TRP superfamily. As a common feature, TRP subfamilies possess a tetrameric structure, each involving six transmembrane domains (S1 to S6) with a highly conservated ankyrin repeat and a ’TRP domain’ [22]. TRPA1 is activated by irritants and pungent agents (e.g., acrolein, AITC) through a reversible covalent modification of specific cysteine residues in the N-terminal [71]. Being an ion channel protein, a pore is formed between the S5 and S6 domains. Molecular modeling and sequence alignment revealed individual residues within the S5 and S6 segments, which are responsible for the opening and activation of the channel [72].

Despite the homology, TRP channels respond to a wide range of stimuli and possess diverse gating mechanisms, ion selectivity, and functions. However, the structural similarities may provide a basis for interaction between distinct TRP receptors when co-expressed [78]. The cooperation between TRPA1 and TRPV1 is suggested by a high-order co-expression of the two TRP receptors. It was earlier described that nearly all TRPA1-sensitive neurons also express TRPV1 [23]. Functional studies proved that the two channels can co-work in a synergistic manner [72,73]. Interestingly, the inflammatory mediator, bradykinin, has been found to activate both TRPA1 and TRPV1, and it seems that both receptors are required for the sensory neurons’ maximum response elicited by bradykinin [74]. The TRPA1 channel activity has been shown to be regulated and modulated by the expression of TPRV1 in trigeminal neurons [79].

Novel research has revealed possible cooperation between TRPA1 and TRPV1 by attaching distinct subunits to create structurally atypical heteromers. Fisher et al. created TRPA1:TRPV1 complexes in cultured sensory neurons. The heteromers that formed were found to be less sensitive to TRPV1 agonists; therefore, it was concluded that TRPV1 activity is inhibited by its combination with TRPA1 subunits [80]. TRPA1 function also appears to be negatively regulated by the complex formation, as TRPA1:TRPV1 heteromers failed to respond to TRPA1 agonists. All TRPs are intended to form tetrameric assemblies, but different TRP subfamilies are able to combine their subunits to form heterotetramer complexes. Although the combination of TRP subunits is not so obvious, the interaction is provided by special modules in the transmembrane domains [81]. The research group also identified a unique domain on the intracellular terminals of TRPV1 that is thought to be responsible for the formation of the TRPA1:TRPV1 complex. The ablation of the identified residues reduced the possibility of the TRPA1:TRPV1 interaction.

**Figure 1:**
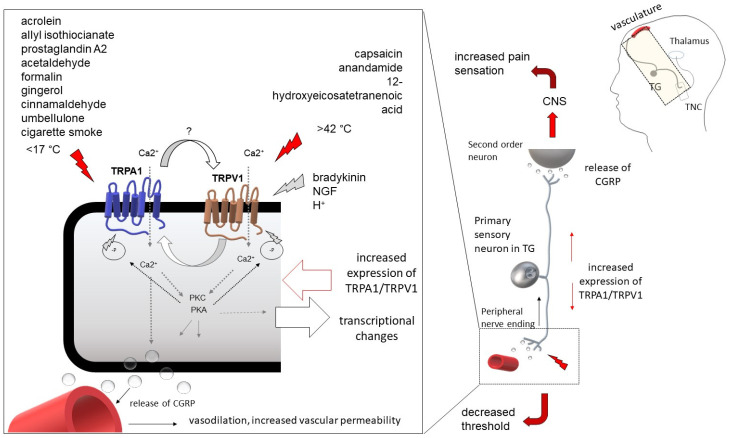
The significance of TRPA1 activation in the development of migraine-related hyperalgesia. The figure shows co-expression of the TRP channels TRPA1 and TRPV1 by primary sensory trigeminal neurons and illustrates a possible interaction between the two channels. Following activation of TRPA1 or TRPV1, heterotetrameric channels can be formed, which may display modified characteristics of both channels, leading to an increase in sensitivity and a decrease in the threshold of the affected neuron. The proposed interaction between TRPs may play a role in migraine-related hypersensitivity.

Tmem100, a conserved transmembrane protein, has been identified as a key regulator of TRPA1:TRPV1 cooperation [82]. This impressive study demonstrated that Tmem100 can physically interact with both TRPA1 and TRPV1 and reduce their association. Tmem100 appears to regulate TRPA1 activity in a TRPV1-dependent manner: in the absence of TRPV1, Tmem100 reduced the activity of the TRPA1. Weng et al. concluded that Tmem100 enhances TRPA1 activity by lowering the potential of TRPV1 to form complexes. They designed a peptide that blocks Tmem100 and consequently inhibits TRPA1 activity. The method could provide specific selective suppression of TRPV1- and TPRA1-mediated pain. The functional significance of the TRPA1:TRPV1 complex in the nociceptive pathway is under ongoing investigation and has not yet been clarified. Such physical interactions between two receptors can alter the receptor properties and excitability, so the cooperation of TRPA1 and TRPV1 should be carefully considered in chronic pain-related disease, such as in migraine, studies (Figure 1).

The TRPA1 channel is also known to form complexes with other ‘pain-related’ receptors, such as the N-methyl-d-aspartate receptor and the delta-opioid receptor [83]. These complexes may also contribute to the modification of the pain sensation and may be involved in the transduction of pain.

### 1.5. Possible Therapeutic Interventions

Supposing the relevant role of TRPA1 in peripheral nociception, the inhibition of TRPA1 appears to be a promising approach in pain therapy.

The first specific TRPA1 antagonists, such as HC–030031, AP–18, and A–967079, were developed and tested more than 10 years ago. Since then, additional TRPA1 antagonists have been developed, and some have also advanced to human trials [84,85,86,87]. However, differences in TRPA1 across species restrict translational research, and most clinical trials have been discontinued due to pharmacokinetic deficiencies; others have yet to be reported.

Desensitization is a well-known and widely studied phenomenon and provides a possible analgesic method in pain research: following initial activation, certain agonists, due to the accumulation of incoming Ca^2+^, may cause the inactivation of the receptor channel and render the channel unresponsive to any further stimulus. This stimulus-evoked desensitization is characteristic of all TRP channels. The therapeutic effect of the desensitization of TRPA1 is supported by a study that showed that safranal, an analgesic plant compound, provides its analgesic effect through the selective desensitization of TRPA1 [88]. Another analgesic peptide, crotalphine, has also been shown to provide its antinociceptive effect through the long-lasting desensitization of TRPA1 [89].

A recent study has validated a human model for assessing TRPA1-mediated pain and demonstrated that the TRPA1 antagonist, (1E,3E)-1-(4-fluorophenyl)-2-methyl-1-penten-3-one oxime (A-967079), is a clinically effective inhibitor of TRPA1 [90].

Focusing on migraine, here we review some migraine medications, which appear to work by the desensitization of the TRPA1. Feverfew (*Tanacetum parthenium*), the well-known anti-migraine agent, exerts its effect through the active ingredient parthenolide. Parthenolide has been shown to act on TRPA1 by inducing its activity-dependent inhibition following its initial activation [91]. Ligustilide (*Angelica sinensis*), another migraine-abortive agent, has also been proven to act as a TRPA1 desensitizing agonist [92].

Extract of butterbur (*Petasites hybridus*), which is used in traditional medicine, is known to have anti-migraine effects. A clinical study confirmed the preventive effect of butterbur in the treatment of migraine [93]. Nearly two decades later, isopetasin, an active component of butterbur, was shown to have a desensitizing effect on TRPA1, which effect is suggested to be responsible for the anti-migraine action [94].

To date, no conclusive evidence exists for the therapeutic efficacy of the administration of TRPA1 antagonists on the inhibition of TRPA1 [95]. However, the proposed therapeutic potential of TRPA1 in headache disorders is supported by the fact that sumatriptan, the iconic anti-migraine medicine, has been recently demonstrated to inhibit TRPA1-mediated vasodilation in meningeal tissues [56].

## 2. Conclusions

In migraine research, it is an outstanding task to explore the sensitization mechanisms of central and peripheral nociceptors. The fact that TRPA1 activation is involved in the trigeminal nociceptive functions has suggested its presumptive role in the development of migraine-related intracranial hypersensitivity. The current data reviewed here indicate that TRPA1 is functionally important in inflammation-induced hyperalgesia in the periphery, which also implies its role in migraine-associated hypersensitivity. Recent studies on TRPA1 exploring the TRPA1-mediated glial–neuronal interactions, the modification of the channel, or its association with other pain-related receptors, provide new insights into the pathomechanism of migraine. According to the reviewed data, the modification of TRPA1 activity in the development of migraine-related hypersensitivity may be considered one of the main directions for migraine research. Furthermore, the data above not only support the putative role of TRPA1 in the pathophysiology of migraine but propose the potency of TRPA1 inhibition in anti-migraine therapy.

## Data Availability

Not applicable.

## References

[1] Burch R.C., Buse D.C., Lipton R.B. (2019). Migraine: Epidemiology, Burden, and Comorbidity. Neurol. Clin..

[2] Solomon S., Cappa K.G., Smith C.R. (1988). Common migraine: Criteria for diagnosis. Headache.

[3] Han S.M., Kim K.M., Cho S.-J., Yang K.I., Kim D., Yun C.-H., Chu M.K. (2021). Prevalence and characteristics of cutaneous allodynia in probable migraine. Sci. Rep..

[4] Ray J.C., Cheema S., Foster E., Gunasekera L., Mehta D., Corcoran S.J., Matharu M.S., Hutton E.J. (2022). Autonomic symptoms in migraine: Results of a prospective longitudinal study. Front. Neurol..

[5] Luda E., Bo E., Sicuro L., Comitangelo R., Campana M. (1991). Sustained visual aura: A totally new variation of migraine. Headache.

[6] Peatfield R.C., Gawel M.J., Rose F.C. (1981). Asymmetry of the aura and pain in migraine. J. Neurol. Neurosurg. Psychiatry.

[7] Russell M.B., Rasmussen B.K., Thorvaldsen P., Olesen J. (1995). Prevalence and sex-ratio of the subtypes of migraine. Int. J. Epidemiol..

[8] Rasmussen B.K., Olesen J. (1992). Migraine with aura and migraine without aura: An epidemiological study. Cephalalgia Int. J. Headache.

[9] Nappi G., Agnoli A., Manzoni G.C., Nattero G., Sicuteri F. (1989). Classification and diagnostic criteria for primary headache disorders (Ad Hoc Committee IHS, 1988). Funct. Neurol..

[10] Lipton R.B., Goadsby P., Silberstein S.D. (1999). Classification and epidemiology of headache. Clin. Cornerstone.

[11] Feindel W., Penfield W., McNaughton F. (1960). The tentorial nerves and Iocalization of intracranial pain in man. Neurology.

[12] Moskowitz M.A. (1984). The neurobiology of vascular head pain. Ann. Neurol..

[13] Moskowitz M.A., Buzzi M.G. (1991). Neuroeffector functions of sensory fibres: Implications for headache mechanisms and drug actions. J. Neurol..

[14] Iizuka T., Sakai F., Suzuki K., Igarashi H., Suzuki N. (2006). Implication of augmented vasogenic leakage in the mechanism of persistent aura in sporadic hemiplegic migraine. Cephalalgia Int. J. Headache.

[15] Albrecht D.S., Mainero C., Ichijo E., Ward N., Granziera C., Zürcher N.R., Akeju O., Bonnier G., Price J., Hooker J.M. (2019). Imaging of neuroinflammation in migraine with aura: A [11C]PBR28 PET/MRI study. Neurology.

[16] Messlinger K. (1997). What is a nociceptor?. Anaesthesist.

[17] Strassman A.M., Raymond S.A., Burstein R. (1996). Sensitization of meningeal sensory neurons and the origin of headaches. Nature.

[18] Kunkler P.E., Zhang L., Pellman J.J., Oxford G.S., Hurley J.H. (2015). Sensitization of the Trigeminovascular System following Environmental Irritant Exposure. Cephalalgia Int. J. Headache.

[19] Caterina M.J., Rosen T.A., Tominaga M., Brake A.J., Julius D. (1999). A capsaicin-receptor homologue with a high threshold for noxious heat. Nature.

[20] Lee H., Iida T., Mizuno A., Suzuki M., Caterina M.J. (2005). Altered thermal selection behavior in mice lacking transient receptor potential vanilloid 4. J. Neurosci. Off. J. Soc. Neurosci..

[21] Bautista D.M., Siemens J., Glazer J.M., Tsuruda P.R., Basbaum A.I., Stucky C.L., Jordt S.-E., Julius D. (2007). The menthol receptor TRPM8 is the principal detector of environmental cold. Nature.

[22] Montell C. (2005). The TRP superfamily of cation channels. Sci. STKE Signal Transduct. Knowl. Environ..

[23] Story G.M., Peier A.M., Reeve A.J., Eid S.R., Mosbacher J., Hricik T.R., Earley T.J., Hergarden A.C., Andersson D.A., Hwang S.W. (2003). ANKTM1, a TRP-like Channel Expressed in Nociceptive Neurons, Is Activated by Cold Temperatures. Cell.

[24] Karashima Y., Talavera K., Everaerts W., Janssens A., Kwan K.Y., Vennekens R., Nilius B., Voets T. (2009). TRPA1 acts as a cold sensor in vitro and in vivo. Proc. Natl. Acad. Sci. USA.

[25] Kobayashi K., Fukuoka T., Obata K., Yamanaka H., Dai Y., Tokunaga A., Noguchi K. (2005). Distinct expression of TRPM8, TRPA1, and TRPV1 mRNAs in rat primary afferent neurons with adelta/c-fibers and colocalization with trk receptors. J. Comp. Neurol..

[26] Jordt S.-E., Bautista D.M., Chuang H.-H., McKemy D.D., Zygmunt P.M., Högestätt E.D., Meng I.D., Julius D. (2004). Mustard oils and cannabinoids excite sensory nerve fibres through the TRP channel ANKTM1. Nature.

[27] Bandell M., Story G.M., Hwang S.W., Viswanath V., Eid S.R., Petrus M.J., Earley T.J., Patapoutian A. (2004). Noxious cold ion channel TRPA1 is activated by pungent compounds and bradykinin. Neuron.

[28] Babes A., Zorzon D., Reid G. (2004). Two populations of cold-sensitive neurons in rat dorsal root ganglia and their modulation by nerve growth factor. Eur. J. Neurosci..

[29] Andersen H.H., Lo Vecchio S., Gazerani P., Arendt-Nielsen L. (2017). Dose-response study of topical allyl isothiocyanate (mustard oil) as a human surrogate model of pain, hyperalgesia, and neurogenic inflammation. Pain.

[30] Kremeyer B., Lopera F., Cox J.J., Momin A., Rugiero F., Marsh S., Woods C.G., Jones N.G., Paterson K.J., Fricker F.R. (2010). A gain-of-function mutation in TRPA1 causes familial episodic pain syndrome. Neuron.

[31] Weyer-Menkhoff I., Pinter A., Schlierbach H., Schänzer A., Lötsch J. (2019). Epidermal expression of human TRPM8, but not of TRPA1 ion channels, is associated with sensory responses to local skin cooling. Pain.

[32] Obata K., Katsura H., Mizushima T., Yamanaka H., Kobayashi K., Dai Y., Fukuoka T., Tokunaga A., Tominaga M., Noguchi K. (2005). TRPA1 induced in sensory neurons contributes to cold hyperalgesia after inflammation and nerve injury. J. Clin. Investig..

[33] Xing J., Lu J., Li J. (2015). TRPA1 mediates amplified sympathetic responsiveness to activation of metabolically sensitive muscle afferents in rats with femoral artery occlusion. Front. Physiol..

[34] Kimball E.S., Prouty S.P., Pavlick K.P., Wallace N.H., Schneider C.R., Hornby P.J. (2007). Stimulation of neuronal receptors, neuropeptides and cytokines during experimental oil of mustard colitis. Neurogastroenterol. Motil. Off. J. Eur. Gastrointest. Motil. Soc..

[35] Grace M., Birrell M.A., Dubuis E., Maher S.A., Belvisi M.G. (2012). Transient receptor potential channels mediate the tussive response to prostaglandin E2 and bradykinin. Thorax.

[36] Bang S., Kim K.Y., Yoo S., Kim Y.G., Hwang S.W. (2007). Transient receptor potential A1 mediates acetaldehyde-evoked pain sensation. Eur. J. Neurosci..

[37] Bautista D.M., Jordt S.-E., Nikai T., Tsuruda P.R., Read A.J., Poblete J., Yamoah E.N., Basbaum A.I., Julius D. (2006). TRPA1 Mediates the Inflammatory Actions of Environmental Irritants and Proalgesic Agents. Cell.

[38] Al-Shamlan F., El-Hashim A.Z. (2019). Bradykinin sensitizes the cough reflex via a B2 receptor dependent activation of TRPV1 and TRPA1 channels through metabolites of cyclooxygenase and 12-lipoxygenase. Respir. Res..

[39] Katsura H., Obata K., Mizushima T., Yamanaka H., Kobayashi K., Dai Y., Fukuoka T., Tokunaga A., Sakagami M., Noguchi K. (2006). Antisense knock down of TRPA1, but not TRPM8, alleviates cold hyperalgesia after spinal nerve ligation in rats. Exp. Neurol..

[40] Tsagareli M.G., Nozadze I., Tsiklauri N., Carstens M.I., Gurtskaia G., Carstens E. (2020). Thermal Hyperalgesia and Mechanical Allodynia Elicited by Histamine and Non-histaminergic Itch Mediators: Respective Involvement of TRPV1 and TRPA1. Neuroscience.

[41] Miyano K., Shiraishi S., Minami K., Sudo Y., Suzuki M., Yokoyama T., Terawaki K., Nonaka M., Murata H., Higami Y. (2019). Carboplatin Enhances the Activity of Human Transient Receptor Potential Ankyrin 1 through the Cyclic AMP-Protein Kinase A-A-Kinase Anchoring Protein (AKAP) Pathways. Int. J. Mol. Sci..

[42] Marcotti A., Fernández-Trillo J., González A., Vizcaíno-Escoto M., Ros-Arlanzón P., Romero L., Vela J.M., Gomis A., Viana F., de la Peña E. (2023). TRPA1 modulation by Sigma-1 receptor prevents oxaliplatin-induced painful peripheral neuropathy. Brain J. Neurol..

[43] Wang S., Qi S., Kogure Y., Kanda H., Tian L., Yamamoto S., Noguchi K., Dai Y. (2021). The ubiquitin E3 ligase Nedd4-2 relieves mechanical allodynia through the ubiquitination of TRPA1 channel in db/db mice. Eur. J. Neurosci..

[44] Shin S.M., Itson-Zoske B., Cai Y., Qiu C., Pan B., Stucky C.L., Hogan Q.H., Yu H. (2020). Satellite glial cells in sensory ganglia express functional transient receptor potential ankyrin 1 that is sensitized in neuropathic and inflammatory pain. Mol. Pain.

[45] Xia M., Chen Y.-J., Chen B., Ru X., Wang J., Lin J., Tang X., Chen W., Hu R., Li W. (2023). Knockout of transient receptor potential ankyrin 1 (TRPA1) modulates the glial phenotype and alleviates perihematomal neuroinflammation after intracerebral hemorrhage in mice via MAPK/NF-κB signaling. Neuroreport.

[46] Demartini C., Greco R., Magni G., Zanaboni A.M., Riboldi B., Francavilla M., Nativi C., Ceruti S., Tassorelli C. (2022). Modulation of Glia Activation by TRPA1 Antagonism in Preclinical Models of Migraine. Int. J. Mol. Sci..

[47] Kunkler P.E., Ballard C.J., Oxford G.S., Hurley J.H. (2011). TRPA1 receptors mediate environmental irritant-induced meningeal vasodilatation. Pain.

[48] Nagata K., Duggan A., Kumar G., García-Añoveros J. (2005). Nociceptor and Hair Cell Transducer Properties of TRPA1, a Channel for Pain and Hearing. J. Neurosci..

[49] Edelmayer R.M., Le L.N., Yan J., Wei X., Nassini R., Materazzi S., Preti D., Appendino G., Geppetti P., Dodick D.W. (2012). Activation of TRPA1 on dural afferents: A potential mechanism of headache pain. Pain.

[50] Huang D., Li S., Dhaka A., Story G.M., Cao Y.-Q. (2012). Expression of the transient receptor potential channels TRPV1, TRPA1 and TRPM8 in mouse trigeminal primary afferent neurons innervating the dura. Mol. Pain.

[51] MacDonald N.J., Butters L., O’Shaughnessy D.J., Riddell A.J., Rubin P.C. (1989). A comparison of the effects of human alpha calcitonin gene-related peptide and glyceryl trinitrate on regional blood velocity in man. Br. J. Clin. Pharmacol..

[52] Goadsby P.J., Edvinsson L. (1993). The trigeminovascular system and migraine: Studies characterizing cerebrovascular and neuropeptide changes seen in humans and cats. Ann. Neurol..

[53] Edvinsson L., Goadsby P.J. (1994). Neuropeptides in migraine and cluster headache. Cephalalgia Int. J. Headache.

[54] Fischer M.J.M., Leffler A., Niedermirtl F., Kistner K., Eberhardt M., Reeh P.W., Nau C. (2010). The General Anesthetic Propofol Excites Nociceptors by Activating TRPV1 and TRPA1 Rather than GABAA Receptors. J. Biol. Chem..

[55] Pozsgai G., Hajna Z., Bagoly T., Boros M., Kemény Á., Materazzi S., Nassini R., Helyes Z., Szolcsányi J., Pintér E. (2012). The role of transient receptor potential ankyrin 1 (TRPA1) receptor activation in hydrogen-sulphide-induced CGRP-release and vasodilation. Eur. J. Pharmacol..

[56] Hansted A.K., Bhatt D.K., Olesen J., Jensen L.J., Jansen-Olesen I. (2019). Effect of TRPA1 activator allyl isothiocyanate (AITC) on rat dural and pial arteries. Pharmacol. Rep. PR.

[57] Wang X.-L., Cui L.-W., Liu Z., Gao Y.-M., Wang S., Li H., Liu H.-X., Yu L.-J. (2019). Effects of TRPA1 activation and inhibition on TRPA1 and CGRP expression in dorsal root ganglion neurons. Neural Regen. Res..

[58] Iversen H.K. (1995). Experimental headache in humans. Cephalalgia Int. J. Headache.

[59] Anderson P.J., Lau G.S., Taylor W.R., Critchley J.A. (1996). Acute effects of the potent lacrimator o-chlorobenzylidene malononitrile (CS) tear gas. Hum. Exp. Toxicol..

[60] Kelman L. (2007). The triggers or precipitants of the acute migraine attack. Cephalalgia Int. J. Headache.

[61] Rozen T.D. (2010). Cluster headache as the result of secondhand cigarette smoke exposure during childhood. Headache.

[62] Brône B., Peeters P.J., Marrannes R., Mercken M., Nuydens R., Meert T., Gijsen H.J.M. (2008). Tear gasses CN, CR, and CS are potent activators of the human TRPA1 receptor. Toxicol. Appl. Pharmacol..

[63] McNamara C.R., Mandel-Brehm J., Bautista D.M., Siemens J., Deranian K.L., Zhao M., Hayward N.J., Chong J.A., Julius D., Moran M.M. (2007). TRPA1 mediates formalin-induced pain. Proc. Natl. Acad. Sci. USA.

[64] Miyamoto T., Dubin A.E., Petrus M.J., Patapoutian A. (2009). TRPV1 and TRPA1 mediate peripheral nitric oxide-induced nociception in mice. PLoS ONE.

[65] Trevisani M., Siemens J., Materazzi S., Bautista D.M., Nassini R., Campi B., Imamachi N., Andrè E., Patacchini R., Cottrell G.S. (2007). 4-Hydroxynonenal, an endogenous aldehyde, causes pain and neurogenic inflammation through activation of the irritant receptor TRPA1. Proc. Natl. Acad. Sci. USA.

[66] Tunis M.M., Wolff H.G. (1952). The hemodynamic analysis of cranial artery pulse wave contours in vascular headache of the migraine type. Trans. Am. Neurol. Assoc..

[67] Kobari M., Meyer J.S., Ichijo M., Imai A., Oravez W.T. (1989). Hyperperfusion of cerebral cortex, thalamus and basal ganglia during spontaneously occurring migraine headaches. Headache.

[68] May A., Büchel C., Turner R., Goadsby P.J. (2001). Magnetic resonance angiography in facial and other pain: Neurovascular mechanisms of trigeminal sensation. J. Cereb. Blood Flow Metab. Off. J. Int. Soc. Cereb. Blood Flow Metab..

[69] Nassini R., Materazzi S., Vriens J., Prenen J., Benemei S., De Siena G., la Marca G., Andrè E., Preti D., Avonto C. (2012). The “headache tree” via umbellulone and TRPA1 activates the trigeminovascular system. Brain J. Neurol..

[70] Jiang L., Wang Y., Xu Y., Ma D., Wang M. (2018). The Transient Receptor Potential Ankyrin Type 1 Plays a Critical Role in Cortical Spreading Depression. Neuroscience.

[71] Zhang L., Kunkler P.E., Knopp K.L., Oxford G.S., Hurley J.H. (2019). Role of intraganglionic transmission in the trigeminovascular pathway. Mol. Pain.

[72] Caterina M.J., Leffler A., Malmberg A.B., Martin W.J., Trafton J., Petersen-Zeitz K.R., Koltzenburg M., Basbaum A.I., Julius D. (2000). Impaired nociception and pain sensation in mice lacking the capsaicin receptor. Science.

[73] Davis J.B., Gray J., Gunthorpe M.J., Hatcher J.P., Davey P.T., Overend P., Harries M.H., Latcham J., Clapham C., Atkinson K. (2000). Vanilloid receptor-1 is essential for inflammatory thermal hyperalgesia. Nature.

[74] Chan C.L.H., Facer P., Davis J.B., Smith G.D., Egerton J., Bountra C., Williams N.S., Anand P. (2003). Sensory fibres expressing capsaicin receptor TRPV1 in patients with rectal hypersensitivity and faecal urgency. Lancet Lond. Engl..

[75] Ogawa N., Kurokawa T., Fujiwara K., Polat O.K., Badr H., Takahashi N., Mori Y. (2016). Functional and Structural Divergence in Human TRPV1 Channel Subunits by Oxidative Cysteine Modification. J. Biol. Chem..

[76] Kumar R., Hazan A., Geron M., Steinberg R., Livni L., Matzner H., Priel A. (2017). Activation of transient receptor potential vanilloid 1 by lipoxygenase metabolites depends on PKC phosphorylation. FASEB J. Off. Publ. Fed. Am. Soc. Exp. Biol..

[77] Robilotto G.L., Mohapatra D.P., Shepherd A.J., Mickle A.D. (2022). Role of Src kinase in regulating protein kinase C mediated phosphorylation of TRPV1. Eur. J. Pain Lond. Engl..

[78] Minke B., Cook B. (2002). TRP Channel Proteins and Signal Transduction. Physiol. Rev..

[79] Salas M.M., Hargreaves K.M., Akopian A.N. (2009). TRPA1-mediated responses in trigeminal sensory neurons: Interaction between TRPA1 and TRPV1. Eur. J. Neurosci..

[80] Fischer M.J.M., Balasuriya D., Jeggle P., Goetze T.A., McNaughton P.A., Reeh P.W., Edwardson J.M. (2014). Direct evidence for functional TRPV1/TRPA1 heteromers. Pflüg. Arch.-Eur. J. Physiol..

[81] Clapham D.E. (2003). TRP channels as cellular sensors. Nature.

[82] Weng H.-J., Patel K.N., Jeske N.A., Bierbower S.M., Zou W., Tiwari V., Zheng Q., Tang Z., Mo G.C.H., Wang Y. (2015). Tmem100 Is a Regulator of TRPA1-TRPV1 Complex and Contributes to Persistent Pain. Neuron.

[83] Cortés-Montero E., Rodríguez-Muñoz M., Ruiz-Cantero M.D.C., Cobos E.J., Sánchez-Blázquez P., Garzón-Niño J. (2020). Calmodulin Supports TRPA1 Channel Association with Opioid Receptors and Glutamate NMDA Receptors in the Nervous Tissue. Int. J. Mol. Sci..

[84] Eid S.R., Crown E.D., Moore E.L., Liang H.A., Choong K.-C., Dima S., Henze D.A., Kane S.A., Urban M.O. (2008). HC-030031, a TRPA1 selective antagonist, attenuates inflammatory- and neuropathy-induced mechanical hypersensitivity. Mol. Pain.

[85] Hu H., Tian J., Zhu Y., Wang C., Xiao R., Herz J.M., Wood J.D., Zhu M.X. (2010). Activation of TRPA1 channels by fenamate nonsteroidal anti-inflammatory drugs. Pflug. Arch..

[86] Chen J., Joshi S.K., DiDomenico S., Perner R.J., Mikusa J.P., Gauvin D.M., Segreti J.A., Han P., Zhang X.-F., Niforatos W. (2011). Selective blockade of TRPA1 channel attenuates pathological pain without altering noxious cold sensation or body temperature regulation. Pain.

[87] Mesch S., Walter D., Laux-Biehlmann A., Basting D., Flanagan S., Miyatake Ondozabal H., Bäurle S., Pearson C., Jenkins J., Elves P. (2023). Discovery of BAY-390, a Selective CNS Penetrant Chemical Probe as Transient Receptor Potential Ankyrin 1 (TRPA1) Antagonist. J. Med. Chem..

[88] Li Puma S., Landini L., Macedo S.J., Seravalli V., Marone I.M., Coppi E., Patacchini R., Geppetti P., Materazzi S., Nassini R. (2019). TRPA1 mediates the antinociceptive properties of the constituent of *Crocus sativus* L., safranal. J. Cell. Mol. Med..

[89] Bressan E., Touska F., Vetter I., Kistner K., Kichko T.I., Teixeira N.B., Picolo G., Cury Y., Lewis R.J., Fischer M.J.M. (2016). Crotalphine desensitizes TRPA1 ion channels to alleviate inflammatory hyperalgesia. Pain.

[90] Heber S., Gold-Binder M., Ciotu C.I., Witek M., Ninidze N., Kress H.-G., Fischer M.J.M. (2019). A Human TRPA1-Specific Pain Model. J. Neurosci. Off. J. Soc. Neurosci..

[91] Materazzi S., Benemei S., Fusi C., Gualdani R., De Siena G., Vastani N., Andersson D.A., Trevisan G., Moncelli M.R., Wei X. (2013). Parthenolide inhibits nociception and neurogenic vasodilatation in the trigeminovascular system by targeting the TRPA1 channel. Pain.

[92] Zhong J., Pollastro F., Prenen J., Zhu Z., Appendino G., Nilius B. (2011). Ligustilide: A novel TRPA1 modulator. Pflug. Arch..

[93] Lipton R.B., Göbel H., Einhäupl K.M., Wilks K., Mauskop A. (2004). Petasites hybridus root (butterbur) is an effective preventive treatment for migraine. Neurology.

[94] Benemei S., De Logu F., Li Puma S., Marone I.M., Coppi E., Ugolini F., Liedtke W., Pollastro F., Appendino G., Geppetti P. (2017). The anti-migraine component of butterbur extracts, isopetasin, desensitizes peptidergic nociceptors by acting on TRPA1 cation channel. Br. J. Pharmacol..

[95] Koivisto A., Jalava N., Bratty R., Pertovaara A. (2018). TRPA1 Antagonists for Pain Relief. Pharmaceuticals.

